# 高通量自动化免疫磁珠净化-超高效液相色谱法检测饲料中4种黄曲霉毒素

**DOI:** 10.3724/SP.J.1123.2022.09006

**Published:** 2023-06-08

**Authors:** Jinnan CHEN, Meng WANG, Zemin DONG, Jin YE, Li LI, Yu WU, Hongmei LIU, Songxue WANG

**Affiliations:** 1.国家粮食和物资储备局科学研究院粮油质量安全研究所, 北京 102629; 1. Institute of Grain and Oil Quality Safety, Academy of National Food and Strategic Reserves Administration, Beijing 102629, China; 2.上海理工大学健康科学与工程学院, 上海 200093; 2. School of Health Science and Engineering, University of Shanghai for Science and Technology, Shanghai 200093, China; 3.江西省农业技术推广中心, 江西 南昌 330046; 3. Jiangxi Agricultural Technology Extension Center, Nanchang 330046, China

**Keywords:** 免疫磁珠, 自动净化, 超高效液相色谱, 黄曲霉毒素, 饲料, immunoaffinity magnetic beads (IMB), automatic purification, ultra performance liquid chromatography (UPLC), aflatoxins, feeds

## Abstract

黄曲霉毒素(AFT)是一种毒性极强的剧毒物质,具有致癌性。饲料原料及产品在生产、运输和储藏等过程存在被黄曲霉毒素污染的风险。该文建立了高通量自动化免疫磁珠净化-超高效液相色谱法测定饲料中4种黄曲霉毒素(黄曲霉毒素B_1_(AFB_1_)、黄曲霉毒素B_2_(AFB_2_)、黄曲霉毒素G_1_(AFG_1_)和黄曲霉毒素G_2_(AFG_2_))的分析方法。饲料样品用乙腈-水(70∶30, v/v)提取,经免疫磁珠自动净化后,使用超高效液相色谱进行分析测试。对磁珠与抗体的偶联比例、免疫磁珠与黄曲霉毒素的反应时间、样品提取液和稀释液等关键实验条件进行了优化,考察了不同饲料样品的净化效果。在优化条件下,豆粕、玉米干酒糟及其可溶物、猪饲料和鸡饲料等4种常见饲料样品在3个水平(5、20和40 μg/kg,以AFB_1_计)下的加标回收率在91.1%~119.4%之间,相对标准偏差小于6.9%;日间精密度为4.5%~7.5%,该方法具有良好的重复性。采用该法检测质量控制样品中AFB_1_的含量,平均值为18.6 μg/kg(*n*=3),准确度为110.3%,测试结果满意。使用该法测试了21种随机购买的饲料样品,其中有4份样品测出含有AFB_1_。本方法所使用的自动净化系统能够自动净化饲料中的4种黄曲霉毒素,并实现样品的批量处理,每批可最多同时净化24个样品,批处理总用时约为30 min;超高效液相色谱法分析速度快,准确性高,可用于检测饲料中黄曲霉毒素的含量。

## Abstract (en)

Aflatoxin (AFT) is an extremely toxic and highly toxic carcinogenic substance. This is particularly problematic due to the risk of aflatoxin contamination in raw feed materials and products during production, transportation, and storage. In this study, immunoaffinity magnetic beads (IMBs) were prepared for the purification of four aflatoxins (aflatoxin B_1_ (AFB_1_), aflatoxin B_2_ (AFB_2_), aflatoxin G_1_ (AFG_1_) and aflatoxin G_2_ (AFG_2_)). The aflatoxin contents were then determined rapidly and accurately using ultra performance liquid chromatography (UPLC). More specifically, the coupling ratio of magnetic beads (MBs) to the aflatoxin monoclonal antibody was initially optimized, wherein an MB volume of 1 mL and an antibody content of 2.0 mg was found to meet the purification requirements of this method. The magnetic properties of the MBs and the IMBs were then investigated using a vibrating sample magnetometer (VSM) at room temperature. As a result, the maximum saturation super magnetizations of the MBs and the IMBs were determined to be 28.61 and 23.22 emu/g, respectively, indicating that the saturation magnetization intensity of the IMBs was reduced by coupling with a non-magnetic antibody. However, the saturation magnetization intensity remained sufficiently high to permit magnetic separation from the solution. In addition, the appearance of the IMBs was examined using a biomicroscope, and it was clear that the magnetic cores were wrapped in agarose gel. Furthermore, the reaction time between the IMBs and the aflatoxins was investigated, and the optimal reaction time for meeting the purification requirements was determined to be 2 min. The stability of the IMBs was then evaluated under refrigerated storage conditions at 4 ℃. It was found that the prepared IMBs maintained a high aflatoxin enrichment capacity for at least eight months. Through the examination of three different extraction solutions, a mixture of acetonitrile and water (70∶30, v/v) was found to be optimal for the extraction of aflatoxins from the feed samples. Moreover, five sample dilutions and purification effects were also examined, and phosphate-buffered saline (containing 0.5% Tween-20) was selected as the preferred sample dilutant. With the optimized conditions, the effectiveness of using IMB for the purification of different feed samples was investigated.

The resulting UPLC chromatogram showed no spurious peaks close to the target peaks, demonstrating a good purification performance. Following matrix spiking (5, 20, and 40 μg/kg, calculated based on AFB_1_) of the four feed samples (i. e., soybean meal, distillers dried grains with solubles, pig feed, and chicken feed), the spiked recoveries of the four aflatoxins ranged from 91.1% to 119.4% with a relative standard deviation (RSD) of <6.9%. In addition, the inter-day precision was 4.5% to 7.5%, and the method exhibited a good reproducibility. Subsequently, the developed method was used to detect AFB_1_ using reference materials. The test value was 18.6 μg/kg with an accuracy of 110.3%, thereby constituting satisfactory results. Upon testing 21 randomly purchased feed samples using this method, four of these samples contained AFB_1_, and the test results obtained using the developed method and stable isotope dilution LC-MS/MS were comparable. It was therefore apparent that the IMB purification method combined with UPLC analysis exhibited a good accuracy for aflatoxin determination. Thus, an automatic purification system was established to facilitate the operation and use of IMBs. This system was able to purify 24 samples simultaneously in 30 min. An IMB purification kit for was also designed and produced for aflatoxin detection in feed samples. The kit contained the sample dilutant, IMBs, the washing solution, and the eluent. After extraction of the feed sample, the extraction solution was added to the sample wells provided in the kit, and the purification system automatically completed the steps of aflatoxin enrichment, impurity washing, and elution of the target toxin. It should be noted that the purification process does not require the operator to manually add the solution, thereby simplifying operation. Overall, the purification method established in this study achieved the high-throughput and automatic purification of the four aflatoxins in feed samples.

黄曲霉毒素(aflatoxins, AFTs)是黄曲霉、寄生曲霉和模式曲霉等在适宜的温、湿度条件下产生的次级代谢产物,极易污染玉米、稻谷、小麦、大豆等农作物^[1⇓⇓⇓-5]^,给畜牧业发展和饲料卫生安全造成极大威胁^[6,7]^。自然条件下产生的黄曲霉毒素主要包括黄曲霉毒素B_1_(AFB_1_)、黄曲霉毒素B_2_(AFB_2_)、黄曲霉毒素G_1_(AFG_1_)和黄曲霉毒素G_2_(AFG_2_),其中AFB_1_毒性最强,污染最广,被世界卫生组织国际癌症研究机构(IARC)确定为Ⅰ类致癌物^[8⇓-10]^。我国颁布的国家标准GB 13078-2017《饲料卫生标准》中规定了各类饲料原料及产品中AFB_1_的限量值,范围为10~50 μg/kg^[11]^。

目前,常用的黄曲霉毒素前处理方法包括免疫亲和柱法^[12,13]^、固相萃取柱法^[14,15]^、QuEChERS^[16,17]^等,上述方法虽然能够有效去除样品中的杂质干扰,但是存在操作复杂、成本高等缺点,尤其是目前最常用的免疫亲和柱法,易出现堵塞柱子的问题,导致样品处理时间长,甚至影响结果的准确性。饲料原料及产品的基质较为复杂,组成也各不相同,前处理方法的净化效果及测定结果会受到一定程度的影响。使用免疫磁珠(IMB)净化真菌毒素的前处理方法已得到了开发并在粮油中得到应用^[18,19]^。免疫磁珠前处理方法基于磁分离技术,能够自动净化处理粮油中的真菌毒素,具有操作简单、批量处理、耗时短和净化效果良好等优点,可减少人工操作带来的误差,提高检测效率及准确性。

高效液相色谱法(HPLC)^[20,21]^、超高效液相色谱法(UPLC)^[22,23]^和液相色谱-串联质谱法(LC-MS/MS)^[24,25]^多用于黄曲霉毒素的检测。LC-MS/MS具有灵敏度高、抗干扰能力强、定量准确等优点,可用于多种真菌毒素的同时检测^[26⇓-28]^。但LC-MS/MS仪器操作复杂,对使用人员要求高,日常维护的成本较高,不适用于基层实验室。使用HPLC检测黄曲霉毒素时需配备衍生器,以增强AFB_1_和AFG_1_的荧光特性,便于检测。另外,HPLC分析速度较慢,溶剂用量大。UPLC基于HPLC的理论及原理,减小色谱柱填料粒径,使用超高压输液泵,提高色谱分离度及灵敏度,分析速度快,同时减少溶剂的使用,降低使用成本^[29]^。

本研究基于免疫磁珠自动净化方法,结合超高效液相色谱的分析技术,建立了一种快速、准确检测饲料原料及产品中黄曲霉毒素的分析方法。本方法能够满足豆粕、玉米干酒糟及其可溶物(DDGS)等饲料原料和鸡、猪等饲料产品中4种黄曲霉毒素的快速、高通量、自动化前处理和高准确性的检测需求。

## 1 实验部分

### 1.1 仪器、试剂与材料

超高效液相色谱仪(Waters Acquity UPLC),配有荧光检测器(FLD),购于美国Waters公司;电子天平(SQP)购于德国Sartorius公司;多管漩涡混合仪(MTV-100)购于杭州奥盛仪器有限公司;离心机(H1850)购于湖南湘仪离心机仪器有限公司;试管旋转混匀仪购于美国Thermo Fisher公司;0.22 μm有机滤膜购于美国Pall公司;振动样品磁强计(VSM, BKT-4500)由北京新科高测科技有限公司提供;正置生物显微镜(UB203i)由重庆澳浦光电技术有限公司提供。

黄曲霉毒素单克隆抗体购于无锡创普生物技术有限公司;磁珠(MB)购于苏州海狸生物医学工程有限公司,表面为*N*-羟基琥珀酰亚胺(NHS)基团修饰,磁珠悬浊液为1 mL磁珠悬浊液中含有200 μL磁珠,粒径为10~30 μm;无水乙醇购于北京化工厂,为分析纯;甲醇、乙腈购于美国Fisher公司,为色谱纯;磷酸盐缓冲液(PBS)盐包购于武汉博士德生物工程有限公司,pH=7.2~7.6;吐温-20购于天津市光复精细化工研究所;吗啉乙磺酸一水合物(MES)购于上海麦克林生化科技有限公司,纯度≥99%;甘氨酸购于北京索莱宝科技有限公司,纯度>99.0%;4种黄曲霉毒素混合溶液(TOXIN-YTBW15,溶剂乙腈)和黄曲霉毒素B_1_溶液标准物质(GBW(E)100302,溶剂甲醇)由国家粮食和物资储备局科学研究院提供,质量控制材料(T04303QC)由英国FAPAS分析实验室能力验证提供,AFB_1_标准值为16.9 μg/kg,范围为9.4~24.3 μg/kg。

### 1.2 免疫磁珠的制备

将磁珠充分混匀,准确吸取1 mL磁珠于离心管中,磁分离后弃去上层液,用无水乙醇快速清洗磁珠2次。用MES缓冲液(100 mmol/L, pH=6.0)稀释2.0 mg的黄曲霉毒素单克隆抗体,混匀后快速加入到清洗完成的磁珠中,在室温下振荡孵育2 h。孵育结束后,磁分离后弃去上层液,加入封闭液(2%甘氨酸),在室温下振荡反应2 h,封闭结束后进行磁分离,然后弃去上层液,使用含0.1%吐温-20的PBS和PBS分别清洗3次。黄曲霉毒素免疫磁珠保存在PBS中,4 ℃冷藏储存。免疫磁珠的制备示意图如图1a所示。

**图1:**
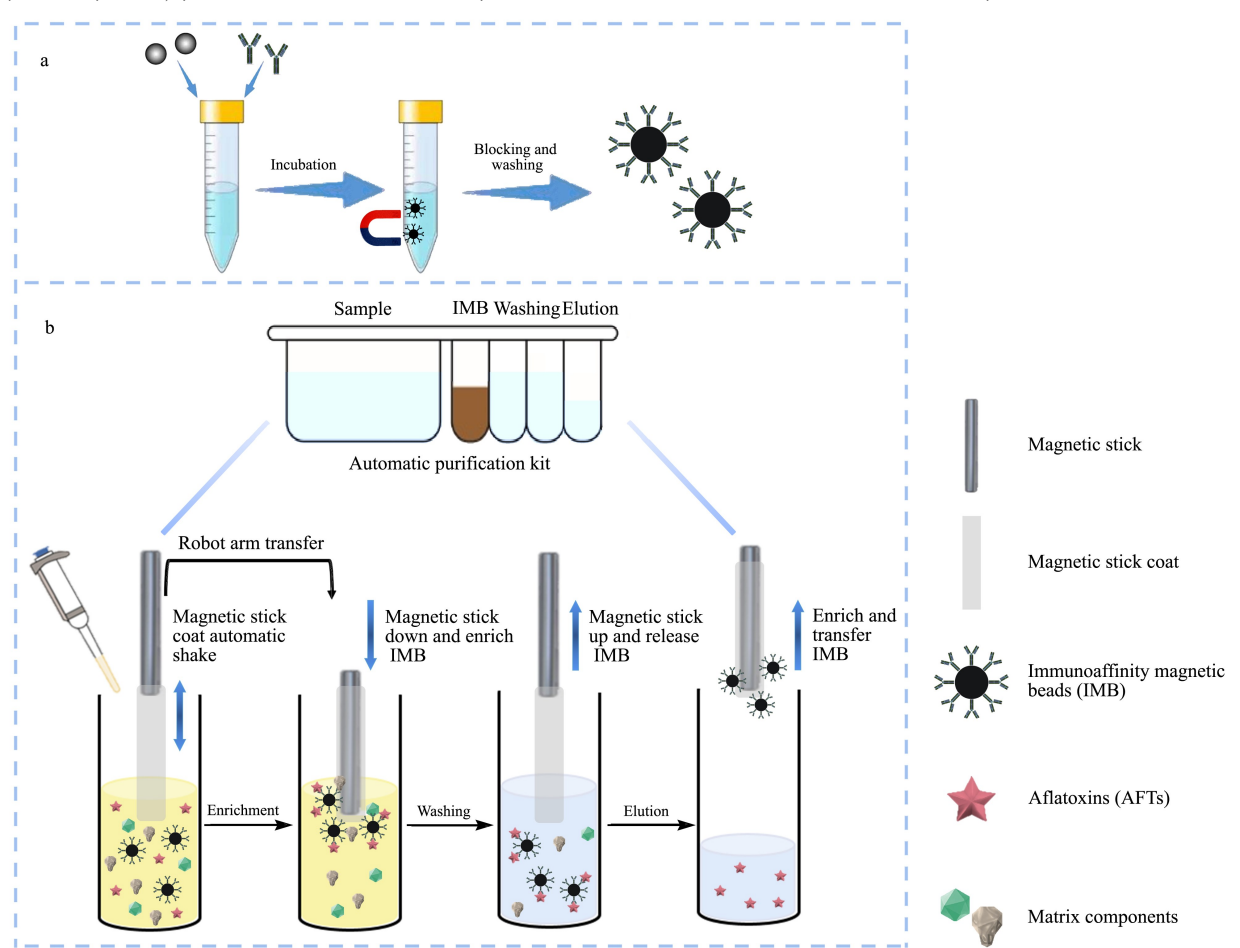
(a)黄曲霉毒素免疫磁珠的制备示意图和(b)免疫磁珠自动净化系统工作流程示意图

### 1.3 样品中黄曲霉毒素的提取和免疫磁珠净化

饲料样品经粉碎、混匀后,准确称取5.00 g(±0.01 g)于50 mL离心管中,加入20 mL乙腈-水(70∶30, v/v),充分涡旋提取20 min,以8000 r/min离心5 min,使固液分离。吸取1 mL上层液移入事先加入含0.5%吐温-20的PBS的试剂盒样品孔中,将试剂盒放到免疫磁珠自动净化系统内进行净化(见图1b)。通过磁棒将100 μL免疫磁珠转移至试剂盒样品孔中,振荡反应2.0 min。通过磁棒将免疫磁珠转移至含1 mL PBS的清洗孔振荡清洗1.0 min,重复两次。通过磁棒将免疫磁珠转移至含0.5 mL甲醇的洗脱孔振荡洗脱1.0 min,最后通过磁棒磁吸后弃去免疫磁珠。净化结束后,向洗脱孔中加入0.5 mL水,混匀后用0.22 μm有机滤膜过滤后收集在进样瓶中,上机检测。

### 1.4 色谱条件

色谱柱:Waters BEH-C_18_色谱柱(100 mm×2.1 mm, 1.7 μm);柱温为40 ℃,样品温度为10 ℃;进样量为10 μL;流动相为甲醇-乙腈-水(17.5∶17.5∶65, v/v/v),等度洗脱;流速为0.2 mL/min。荧光检测器的激发波长为360 nm,发射波长为440 nm。

## 2 结果与讨论

### 2.1 免疫磁珠的合成与表征

磁珠上抗体的偶联量是免疫磁珠制备的关键点之一。若抗体的偶联量不足,免疫磁珠富集目标毒素的容量较低,无法满足检测要求;若制备过程加入的抗体过多,过量的抗体无法偶联到磁珠上,会导致抗体浪费,成本提高。本实验优化了磁珠与抗体的偶联量,1 mL磁珠分别与2.0、2.5、3.0和4.0 mg黄曲霉毒素单克隆抗体结合,计算加标回收率(加标水平为1.25 μg/kg,以AFB_1_计)及100 μL免疫磁珠的最大吸附量(即免疫磁珠的容量),结果如图2a所示。随着抗体偶联量的增加,每100 μL免疫磁珠的容量也随之增加,由125.6 ng增加至202.6 ng。AFB_1_、AFB_2_、AFG_1_和AFG_2_的加标回收率在94.3%~102.8%之间,RSD小于8.3%,均满足测试要求。考虑到抗体的成本及饲料中黄曲霉毒素的限量,选择每毫升磁珠中含有黄曲霉毒素单克隆抗体2.0 mg作为饲料中黄曲霉毒素的免疫磁珠偶联比例。

**图2:**
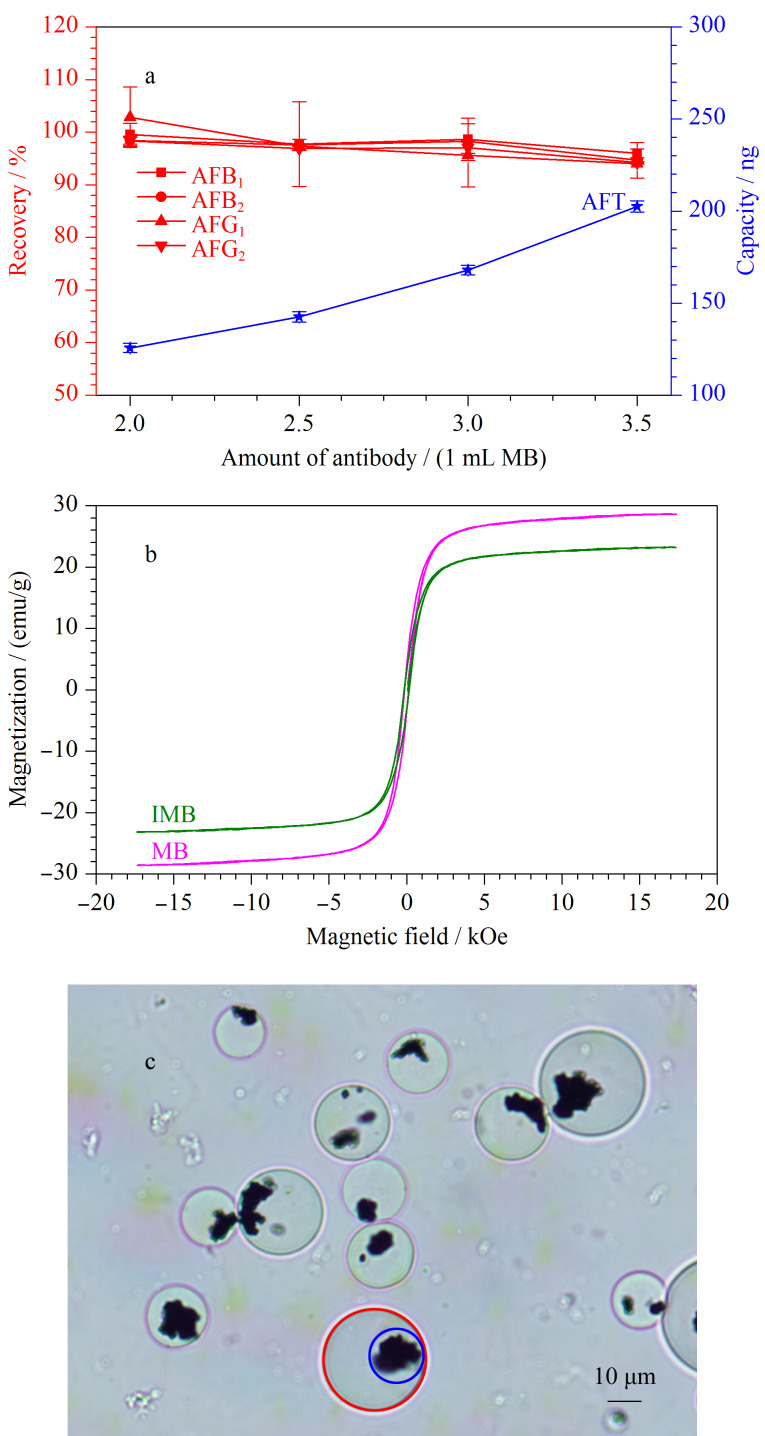
(a)磁珠与不同量的抗体偶联的AFTs加标回收率(*n*=3)及最大吸附量、(b)MB和IMB的常温VSM图和(c)IMB的显微镜图

磁珠的磁性在磁分离中起到关键作用,磁性会影响本方法的准确性。使用VSM测量了MB和IMB的磁性,如图2b所示。MB和IMB的饱和磁化强度分别为28.61和23.22 emu/g。尽管偶联了非磁性的抗体使IMB的饱和磁化强度有所下降,但其饱和磁化强度仍较高,足以从溶液中进行磁分离。去掉外磁场时,IMB通过简单混匀仍可以均匀地分散在溶液中。本文所使用的磁珠是以天然的亲水性高分子琼脂糖为基础的多孔微球,表面修饰了NHS基团。使用生物显微镜(目镜放大倍数为10倍,物镜放大倍数为60倍)对偶联黄曲霉毒素单克隆抗体后的磁珠进行表征,如图2c所示,红色线内为琼脂糖微球,蓝色线内为磁核,琼脂糖微球直径约为15~30 μm。

### 2.2 免疫磁珠稳定性的考察

为考察黄曲霉毒素免疫磁珠在4 ℃冷藏储存条件下的稳定性,分别在第0(免疫磁珠合成当天)、2、4、6和8个月对黄曲霉毒素混合溶液(加标水平为1.25 μg/kg,以AFB_1_计)进行测试,结果如图3所示。在第0~8个月的时间内,黄曲霉毒素免疫磁珠的加标回收率为88.2%~106.7%, RSD小于8.5%,测试结果有轻微波动,但是整体保持稳定水平。因此,黄曲霉毒素免疫磁珠在4 ℃冷藏储存条件下,至少可以在8个月内对目标毒素保持较高的富集能力。

**图3:**
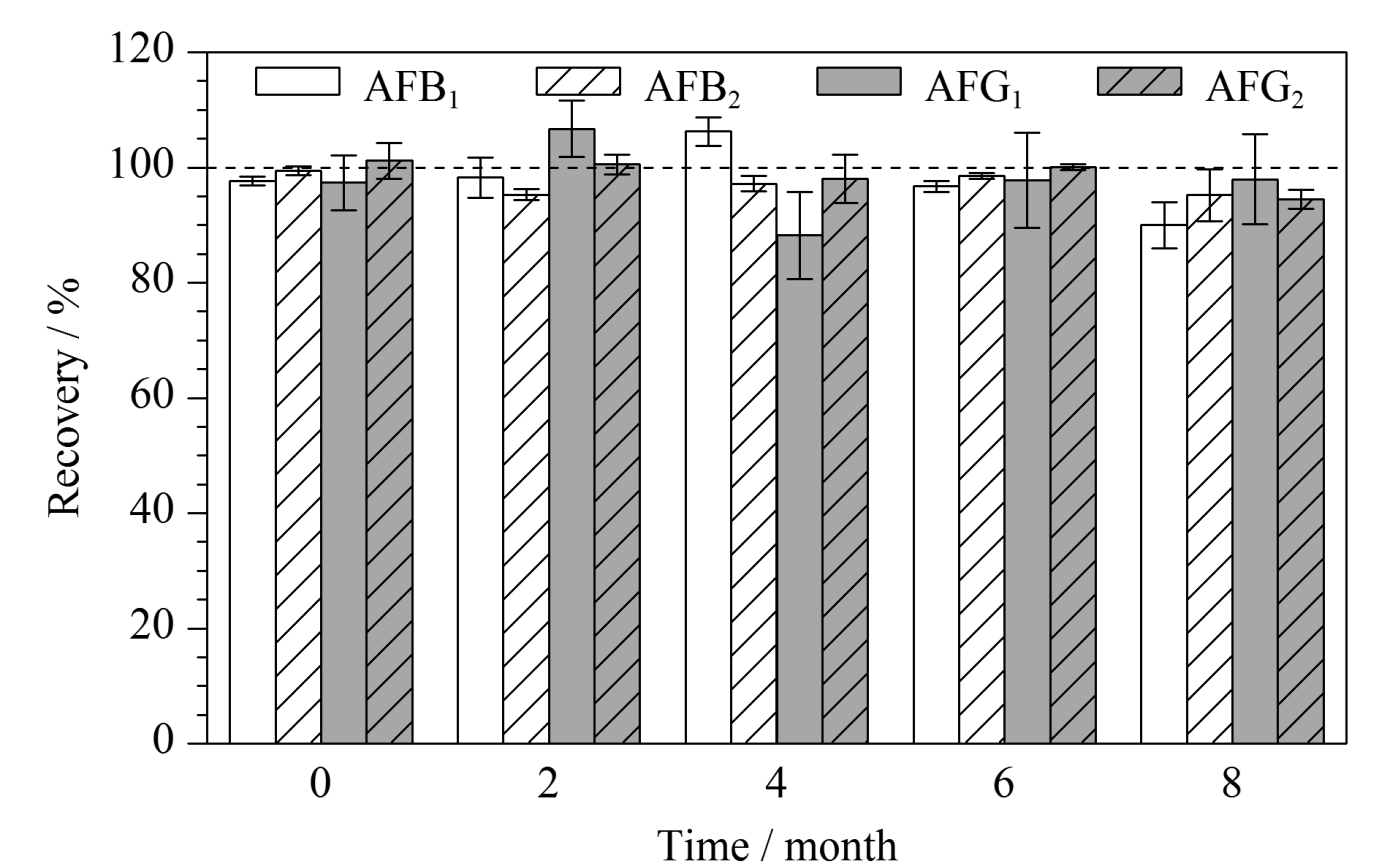
黄曲霉毒素免疫磁珠的稳定性测试结果(*n*=3)

### 2.3 免疫磁珠自动净化系统

通过本研究团队前期开发的免疫磁珠自动净化系统^[30]^,利用磁珠良好的超顺磁性,采用外部磁场(磁棒)和磁棒套的有效控制对免疫磁珠进行富集和释放。通过连接磁棒和磁棒套的水平和垂直移动机械臂可以将磁棒套外富集的免疫磁珠转移到各个孔位中。当磁棒和磁棒套分离时,外加磁场消失,使免疫磁珠从磁棒套释放到各孔溶液中。图1b显示了饲料中黄曲霉毒素免疫磁珠自动净化系统的工作流程。饲料样品经提取后加入到样品孔中,该系统将自动完成样品中黄曲霉毒素的富集、杂质清洗和洗脱等净化步骤,每批可最多同时净化24个样品,批处理总用时约为30 min。为配合免疫磁珠自动净化系统的使用,本研究团队设计和制作了饲料中黄曲霉毒素免疫磁珠净化试剂盒,内置了样品稀释液、免疫磁珠、清洗液和洗脱液,无需实验人员手动添加,使操作更加简单、方便,可进行批量处理,实现样品的高通量、自动净化,提高了检测效率。

### 2.4 方法条件的优化

#### 2.4.1 反应时间的优化

足够的反应时间能够保证免疫磁珠上的抗体与黄曲霉毒素充分结合,但又需要缩短反应时间以提高前处理效率。将免疫磁珠与黄曲霉毒素混合溶液(加标水平为5 μg/kg,以AFB_1_计)反应,考察不同反应时间(0.5、2.0、4.0、6.0和8.0 min)下免疫磁珠与黄曲霉毒素的结合效率。结果如图4所示,反应2.0 min后免疫磁珠与黄曲霉毒素的结合效率可达100%以上,且随着时间的增加,回收率基本不变。说明2.0 min的反应时间可以满足黄曲霉毒素免疫磁珠的测试要求。

**图4:**
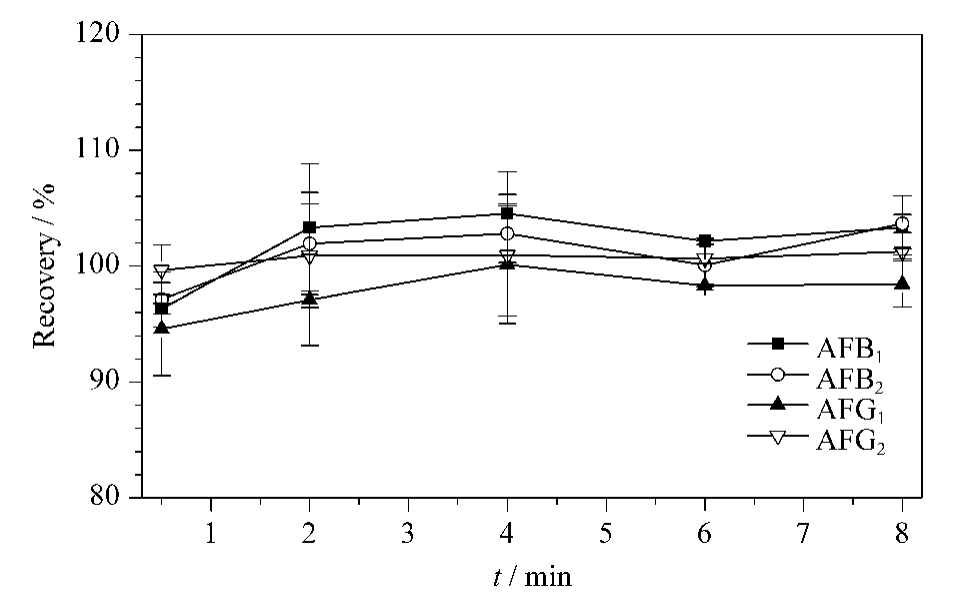
在不同反应时间下免疫磁珠与黄曲霉毒素的结合效率(*n*=3)

#### 2.4.2 样品提取液的优化

由于饲料成分较为复杂,选择合适的提取液对提高样品的回收率具有重要作用。黄曲霉毒素具有较高的极性,易溶解于极性或者极性稍强的有机溶剂,如甲醇、乙腈等^[10,31]^。本文考察了甲醇-水(70∶30, v/v)、乙腈-水(70∶30, v/v)和乙腈-水(84∶16, v/v)3种提取液对饲料中黄曲霉毒素提取效果的影响。采用加标回收的方式验证,添加黄曲霉毒素混合溶液(加标水平为20 μg/kg,以AFB_1_计)于DDGS阴性样品中,加入不同的提取液进行提取,测试结果如图5所示。甲醇-水(70∶30, v/v)作为提取液时,提取效果不佳,回收率仅为60.5%~63.8%。乙腈-水(70∶30, v/v)和乙腈-水(84∶16, v/v)的提取效率较高,回收率分别为94.5%~100.3%和93.7%~99.6%之间,RSD分别小于4.4%和6.3%。考虑到有机溶剂用量,选择乙腈-水(70∶30, v/v)作为饲料中黄曲霉毒素的提取液。

**图5:**
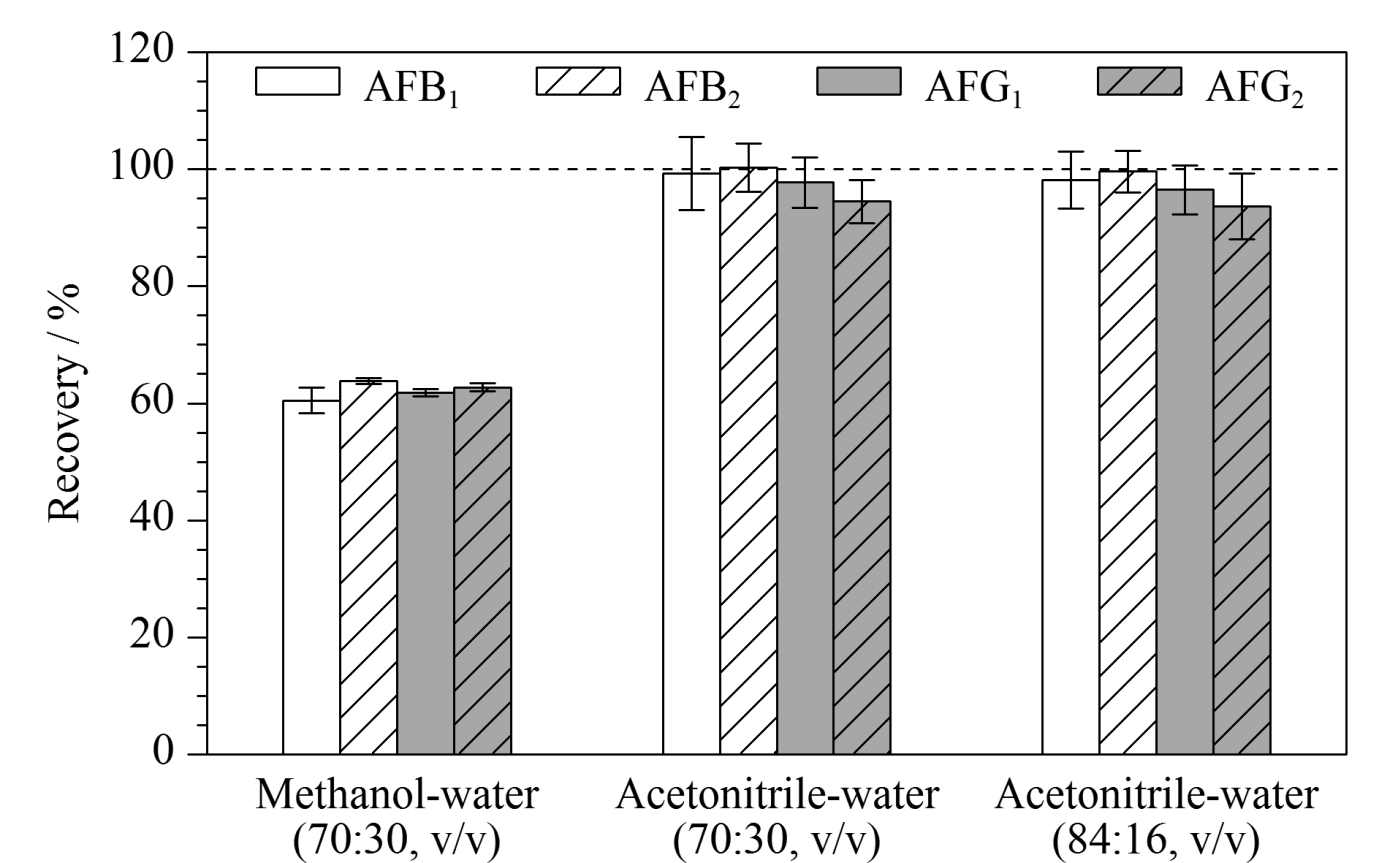
不同提取液对黄曲霉毒素回收率的影响(*n*=3)

#### 2.4.3 样品稀释液的优化

免疫磁珠上的抗体对有机溶剂的耐受性是有限的。样品经过乙腈-水(70∶30, v/v)提取后,需要对提取液进行稀释才可进行免疫磁珠净化,以确保抗体的活性。选取DDGS阴性样品进行提取液加标实验(加标水平为1.25 μg/kg,以AFB_1_计),使用含不同体积分数(0.1%、0.3%、0.5%、0.75%和1.0%)吐温-20的PBS对样品提取液进行稀释。结果显示含不同体积分数吐温-20的PBS未明显影响磁珠性能(如图6a),加标回收率在86.0%~100.7%之间,RSD小于6.2%。但是,当吐温-20体积分数为0.1%和0.3%时,样品提取液和稀释液在混合过程中会出现絮状物杂质,影响净化效果。而含有0.5%及以上的吐温-20时,无絮状物杂质,能够有效减少饲料提取液中杂质的干扰,同时保证目标毒素不损失。因此,选择含有0.5%吐温-20的PBS作为饲料样品的稀释液,豆粕、DDGS、鸡饲料和猪饲料等样品经免疫磁珠净化后的超高效液相色谱图如图6b,不同饲料基质的净化效果较好,目标峰附近均无杂峰。

**图6:**
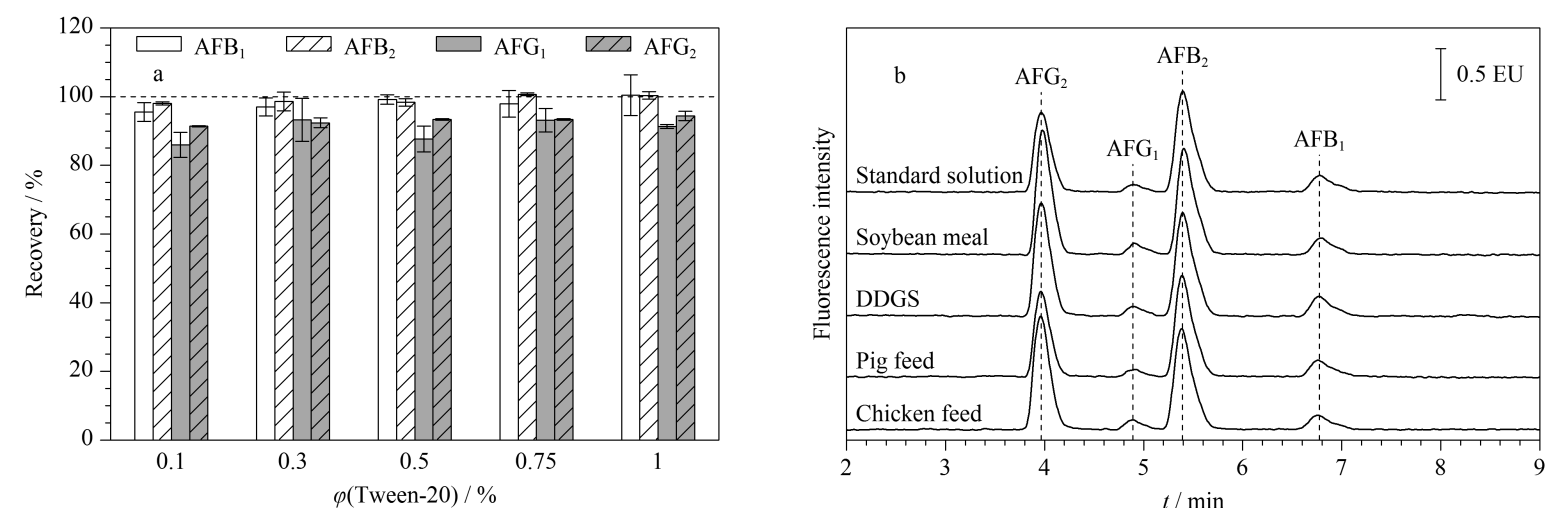
(a)不同稀释液对AFTs回收率的影响(*n*=3)和(b)免疫磁珠净化的不同饲料样品的UPLC色谱图

### 2.5 方法评价

#### 2.5.1 线性范围和检出限

在1.4节色谱条件下,测定黄曲霉毒素混合溶液(0.625、1.25、2.5、5、10和20 μg/kg,以AFB_1_计),以测得的峰面积为纵坐标,相应的质量浓度为横坐标绘制标准曲线,以3倍信噪比(*S/N*)计算仪器的检出限,10倍信噪比计算仪器的定量限。在1.3节前处理条件下,对样品进行了4倍稀释。结果表明,AFB_1_、AFB_2_、AFG_1_和AFG_2_分别在2.5~80、0.625~20、2.5~80和0.625~20 μg/kg范围内呈线性关系,相关系数(*R*^2^)分别为0.9998、0.9999、0.9999和0.9999。根据样品稀释倍数,计算得到方法的检出限分别为2.44、0.08、2.52和0.08 μg/kg,方法的定量限分别为8.16、0.24、8.40和0.20 μg/kg,可以满足日常检测要求。

#### 2.5.2 准确度和精密度

考察了用黄曲霉毒素免疫磁珠净化豆粕、DDGS、猪饲料和鸡饲料等4种饲料在低、中、高(5、20和40 μg/kg,以AFB_1_计)3个水平下的基质加标回收率,测试结果见表1。豆粕、DDGS、猪饲料和鸡饲料的加标回收率在91.1%~119.4%之间,RSD小于6.9%。选取DDGS作为基质加标样品(20 μg/kg,以AFB_1_计),使用免疫磁珠净化,重复测定3天,每天测2次,考察方法的日间精密度,测试结果为4.5%~7.5%,表明该方法具有良好的重复性。该方法的准确性也通过使用FAPAS质量控制材料(T04303QC)进行了验证,获得的AFB_1_含量的平均值为18.6 μg/kg(*n*=3), RSD为0.9%,准确度为110.3%。

**表1:** 不同饲料样品中黄曲霉毒素的加标回收率和RSD(*n*=3)

Substrate	Spiked level/ (μg/kg)	AFB_1_			AFB_2_			AFG_1_			AFG_2_		
		Recovery/%	RSD/%		Recovery/%	RSD/%		Recovery/%	RSD/%		Recovery/%	RSD/%	
Soybean	5	99.7	4.8		108.8	2.4		100.1	6.6		104.7		3.2
meal	20	98.3	1.2		109.9	1.4		98.4	0.5		104.8		2.0
	40	95.4	1.4		107.0	0.9		95.2	1.7		99.8		1.3
DDGS	5	119.4	5.4		113.2	1.5		94.7	1.4		103.7		0.8
	20	99.1	0.7		105.5	0.4		92.8	1.6		100.6		0.5
	40	94.7	0.6		103.1	1.4		92.2	2.4		97.6		1.4
Pig feed	5	116.0	3.9		116.8	2.0		112.3	1.9		107.2		4.9
	20	112.3	1.4		114.0	1.8		105.8	2.2		112.9		1.9
	40	110.7	3.3		113.8	2.7		110.2	2.0		113.8		1.6
Chicken	5	91.9	6.9		112.5	1.9		93.2	2.0		105.9		1.7
feed	20	97.9	3.1		114.8	1.1		94.8	1.0		105.6		1.5
	40	92.1	0.8		110.2	1.0		91.1	2.1		99.3		0.2

#### 2.5.3 实际样品测试

随机购买21份样品,包含豆粕、DDGS等饲料原料,鸡、猪、羊、牛等饲料产品。采用本研究建立的免疫磁珠自动净化-超高效液相色谱法对21份实际样品中4种黄曲霉毒素进行检测。结果显示(表2),有4份样品检出AFB_1_,检出率为19.0%,超标率为9.5%, 未检出AFB_2_、AFG_1_和AFG_2_。采用稳定同位素稀释-液相色谱-串联质谱法^[32]^对上述21份样品进行复测,两种检测方法得到的测试结果相当,说明免疫磁珠自动净化-超高效液相色谱法具有良好的准确性。选取饲料阳性样品,并对此样品进行AFB_1_加标测试,从两种检测黄曲霉毒素方法的液相色谱图中可以看出(图7),稳定同位素稀释法的净化效果较差,杂峰较多;免疫磁珠的净化效果较好,目标峰附近无杂峰。

**表2:** 两种检测方法对21种饲料样品的测试结果

No.	Feed	AFB_1_/(μg/kg)		No.	Feed	AFB_1_/(μg/kg)		No.	Feed	AFB_1_/(μg/kg)		
		This method	Ref. [32]			This method	Ref. [32]			This method	Ref. [32]	
1	chicken feed	18.0	20.6	8	sheep feed	<LOD	<LOD	15	cattle feed	<LOD		<LOD
2	chicken feed	38.6	41.9	9	cattle feed	<LOD	<LOD	16	cattle feed	5.1		6.2
3	chicken feed	<LOD	<LOD	10	cattle feed	<LOD	<LOD	17	cattle feed	<LOD		<LOD
4	sheep feed	<LOD	<LOD	11	cattle feed	<LOD	<LOD	18	cattle feed	<LOD		<LOD
5	sheep feed	7.3	9.5	12	cattle feed	<LOD	<LOD	19	pig feed	<LOD		<LOD
6	sheep feed	<LOD	<LOD	13	cattle feed	<LOD	<LOD	20	soybean meal	<LOD		<LOD
7	sheep feed	<LOD	<LOD	14	cattle feed	<LOD	<LOD	21	DDGS	<LOD		<LOD

**图7:**
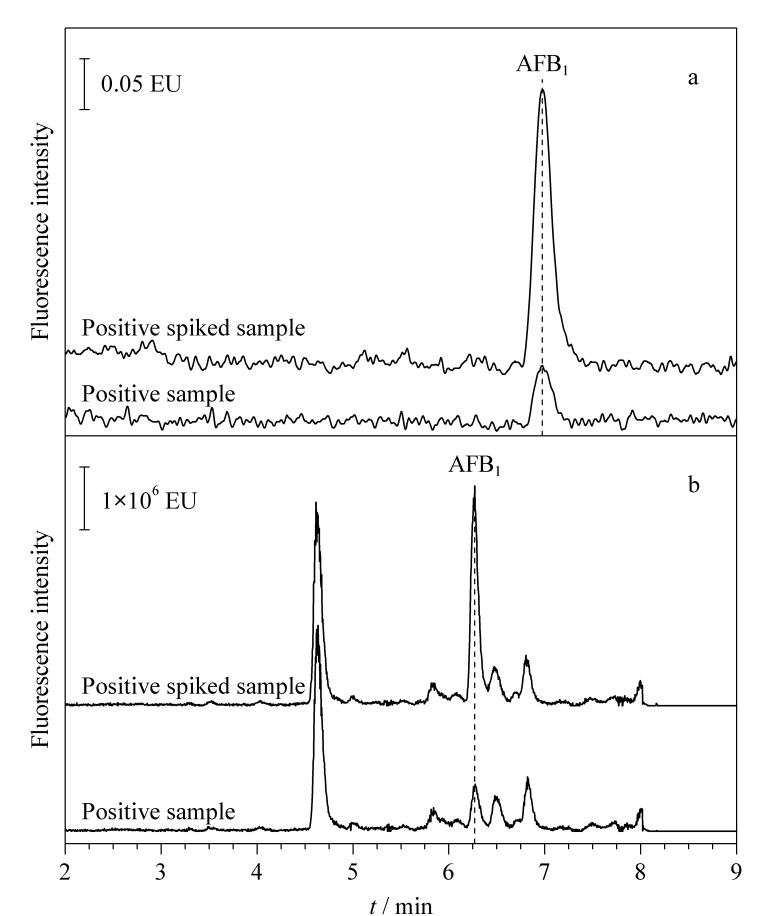
采用(a)本方法和(b)稳定同位素稀释-LC-MS/MS 检测阳性样品及阳性加标样品时的色谱图

## 3 结论

本文开发了免疫磁珠自动净化技术,结合超高效液相色谱分析方法,用于检测饲料中4种黄曲霉毒素。对磁珠与抗体的偶联比例、反应时间、样品提取液和稀释液等条件进行了优化。本方法实现了饲料中黄曲霉毒素的高通量、全自动净化处理,可有效降低实验人员的操作难度,提高真菌毒素检测效率。验证结果显示,该方法具有较为满意的线性范围、标准曲线、检出限、定量限、准确性及重复性,且净化效果较好,可满足饲料原料及不同动物饲料产品中4种黄曲霉毒素的实际检测。
